# Machine learning in paediatric haematological malignancies: a systematic review of prognosis, toxicity and treatment response models

**DOI:** 10.1038/s41390-024-03494-9

**Published:** 2024-08-31

**Authors:** Gerard Gurumurthy, Juditha Gurumurthy, Samantha Gurumurthy

**Affiliations:** 1 School of Medicine, University of Manchester, Manchester, UK; 2 School of Cancer and Pharmaceutical Sciences, King’s College London, London, UK; 3 Department of Infectious Diseases & Immunology, Imperial College London, London, UK

## Abstract

**Background:**

Machine Learning (ML) has demonstrated potential in enhancing care in adult oncology. However, its application in paediatric haematological malignancies is still emerging, necessitating a comprehensive review of its capabilities and limitations in this area.

**Methods:**

A literature search was conducted through Ovid. Studies included focused on ML models in paediatric patients with haematological malignancies. Studies were categorised into thematic groups for analysis.

**Results:**

Twenty studies, primarily on leukaemia, were included in this review. Studies were organised into thematic categories such as prognoses, treatment responses and toxicity predictions. Prognostic studies showed AUC scores between 0.685 and 0.929, indicating moderate-high predictive accuracy. Treatment response studies demonstrated AUC scores between 0.840 and 0.875, reflecting moderate accuracy. Toxicity prediction studies reported high accuracy with AUC scores from 0.870 to 0.927. Only five studies (25%) performed external validation. Significant heterogeneity was noted in ML tasks, reporting formats, and effect measures across studies, highlighting a lack of standardised reporting and challenges in data comparability.

**Conclusion:**

The clinical applicability of these ML models remains limited by the lack of external validation and methodological heterogeneity. Addressing these challenges through standardised reporting and rigorous external validation is needed to translate ML from a promising research tool into a reliable clinical practice component.

**Impact:**

- Key message: Machine Learning (ML) significantly enhances predictive models in paediatric haematological cancers, offering new avenues for personalised treatment strategies. Future research should focus on developing ML models that can integrate with real-time clinical workflows.
- Addition to literature: Provides a comprehensive overview of current ML applications and trends. It identifies limitations to its applicability, including the limited diversity in datasets, which may affect the generalisability of ML models across different populations.
- Impact: Encourages standardisation and external validation in ML studies, aiming to improve patient outcomes through precision medicine in paediatric haematological oncology.

## Introduction

Machine Learning (ML), a subset of artificial intelligence, is capable of identifying complex patterns within large datasets. By leveraging advanced algorithms, ML can facilitate significant advancements in diagnostics, prognostics, and therapeutic decision-making. Despite its potential, the application of ML in healthcare remains largely limited to adult oncology, radiology, and pathology, where it has shown promise in enhancing diagnostic accuracy and treatment planning.^1–4^ However, its utilisation in paediatric haematological malignancies is still in its infant stages, primarily due to the unique challenges and complexities associated with paediatric cancers.

Paediatric haematological cancers present an area where ML can be beneficially utilised. Children with haematological malignancies exhibit diverse biological behaviours and responses to treatment, necessitating highly individualised therapeutic approaches.^5^ The heterogeneity of these diseases, coupled with the varying responses to existing therapies, underscores the need for a nuanced approach that balances effective treatment with the minimisation of long-term adverse effects.^6–8^ ML, with its ability to process and analyse vast amounts of data, offers the potential to develop more precise and personalised treatment strategies, thereby improving prognosis and reducing treatment-related toxicity in paediatric patients.

The European Union’s Beating Cancer Plan underscores the importance of integrating advanced technologies, including ML, into cancer care.^9^ This initiative aims to exploit the predictive/ classification power of ML to enhance cancer prevention, diagnosis, and treatment across Europe. In the context of paediatric haematological malignancies, the potential benefits of ML are particularly significant. The ability to predict disease progression, treatment response, and adverse effects with greater accuracy can transform clinical care, enabling more targeted and effective interventions. It is therefore necessary to address the current limitations of ML, including the need for diverse and representative datasets, standardised reporting, and rigorous external validation. This systematic review aims to provide a comprehensive overview of the current applications of ML in paediatric haematological malignancies, assessing its potential to enhance diagnostic accuracy, prognostic predictions, and treatment strategies.

## Methods

This review was conducted in accordance with the Preferred Reporting Items for Systematic reviews and Meta-Analyses (PRISMA) guidelines and was registered with PROSPERO (CRD42024507811). A comprehensive systematic review of the literature was carried out in February 2024 using the OVID platform. The databases searched included AMED, EMBASE, MEDLINE, and Emcare. The detailed search strategy is provided in Supplementary Table S1.

### Inclusion and exclusion criteria

To be included in the review, studies had to focus on the application of ML in paediatric haematological cancers, detailing the type of ML model and its methodology. Only original research articles were considered; reviews, case reports, and other non-original research articles were excluded. Additionally, only studies exclusively involving paediatric populations were included; mixed studies with both paediatric and adult cohorts were excluded. Articles were limited to those published in English.

### Data extraction and synthesis

Analysis of records were conducted by two authors independently. Data extracted from each study included the specific type of haematological cancer investigated, the tasks performed by the ML program, the number of patients involved, the ML method employed, input and output variables, the method of cross-validation used, and any external validation performed. Studies were then grouped based on their primary objectives or outcomes related to paediatric haematological cancers, such as prognosis, treatment response, and toxicity models. This thematic grouping facilitated a narrative synthesis to highlight trends, patterns, and gaps in the current research. A minimum of three studies was required to synthesise a theme, ensuring sufficient data to capture the scope and trends of current research efforts.

### Quality assessment

The quality of the studies was assessed using appropriate tools. For studies investigating prognostic ML models, the Quality in Prognosis Studies (QUIPS)^10^ checklist was utilised. For all other thematic groups, the Newcastle-Ottawa Scale (NOS)^11^ was used to assess the quality, given the observational nature of the included studies.

### Analysis

Due to the varying nature of the ML tasks, lack of uniform reporting formats, and diverse effect measures, formal meta-analyses were deemed unfeasible. Instead, heterogeneity was addressed qualitatively by describing differences in study populations, methodologies, outcomes, and effect measures.

## Results

**Fig. 1:**
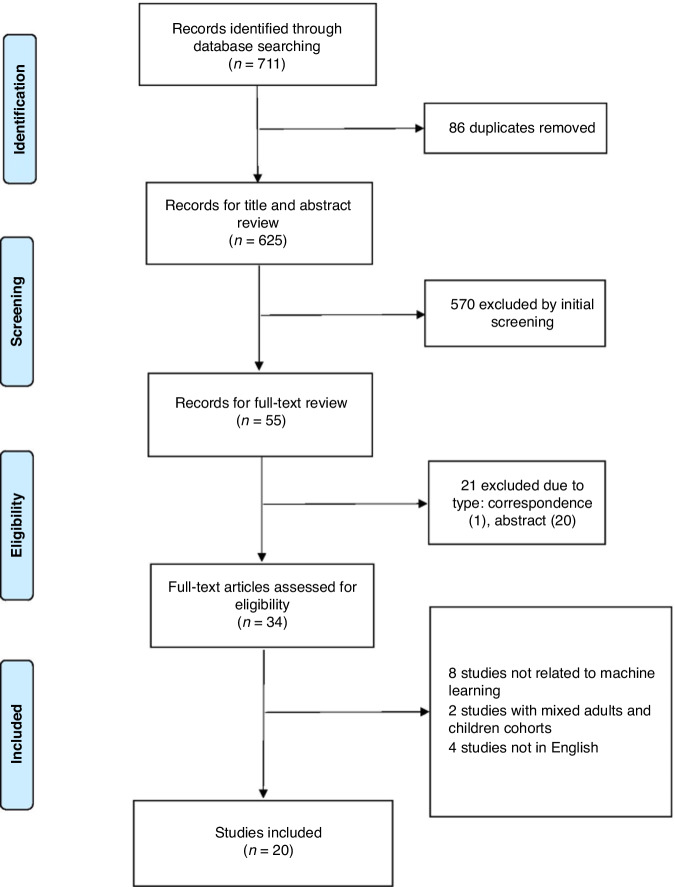
Flow diagram of studies shortlisted for inclusion in this review from the initial search results (*n* = 20).

Searches conducted through the available databases in Ovid yielded a total of 711 results (Fig. 1). Of which, 20 studies that applied ML in paediatric haematological malignancies met the inclusion criteria for this review (Table 1).^12–31^

**Table 1:** Characteristics of Included Studies Exploring Machine Learning in Paediatric Haematological Malignancies.

Study	Cancer Type	Task/ Problem	Input Variable	Output Variable	ML Method	No. of Patients	Cross validation	External Validation	Highest AUC	Other Comparative Statical Scores Used
**Prognosis and Relapse/ Recurrence Studies**										
He et al. ^12^	AML	Prognostic prediction	Expression levels of pyroptosis-related genes	Risk score	LASSO	NA	Not specified	E-MTAB-1216 dataset	0.893	HR = 2.04
He et al. ^13^	ALL	Survival prediction	Clinical characteristics, immunophenotype, genetic data	EFS predictive model	LASSO	1693	10-fold CV	Not specified	0.822	C-index = 0.81
Cui et al. ^14^	Leukaemia (sub-type not specified)	Survival prediction	Clinical characteristics	Survival predictive model	Bayesian inference	17539	Not specified	Not specified		C-index = 0.93
Zheng et al. ^15^	AML	Prognostic prediction	Gene expression data of m6A-related lncRNAs	OS predictive model	LASSO	646	Not specified	TCGA database	0.685	C-index = 0.82
Bohannan et al. ^16^	ALL	Survival prediction	Genomic data	EFS predictive model	RF	156	Training (70%) and testing (30%) cohorts used	Not specified	0.929	HR = 5.41 C-index = 0.82
Gao et al. ^17^	B-cell ALL	Survival prediction	Clinical characteristics	OS predictive model	LASSO	1316	C-index	TARGET database	0.898	C-index = 0.87
Lin et al. ^18^	B-cell ALL	Relapse prediction	NAD+ metabolism-related genes	Relapse predictive model	RF	NA	Mentioned but not specified	Not specified	0.8031	
Pan et al. ^19^	ALL	Relapse prediction	Sociodemographic, clinical, immunological, and cytogenetic data	Relapse predictive model	RF, SVM, LR & DT	570	10-fold CV	Independent test set of 84 patients	0.904	Accuracy = 82.9%
**Treatment Response Studies**										
Gbadamosi et al. ^20^	AML	GO-related response prediction	Genetic data	Treatment response outcome model	LASSO	301	1000-fold CV	Not specified		OS = 0.676 HR = 0.565
Pedreira et al. ^21^	ALL	Treatment intensity decision support model	Clinical data	Treatment decision model	NN	158	Leave-one-out	Not specified		RHR = 98% ROR = 21% RSR = 0%
Gal et al. ^22^	AML	Complete remission prediction	Gene expression data	Complete remission predictive model	K-NN, SVM & RF	473	5-fold CV	Not specified	0.840	
Kashef et al. ^23^	ALL	Treatment prediction	Clinical data	Complete remission predictive model	GBM, RF, GLM	241	5-fold CV	Not specified	0.8725	
Kashef et al. ^24^	ALL	Treatment outcome classification	Clinical characteristics & treatment related toxicity	Classification of treatment outcomes model	DT, SVM, RF,LDA,MLR, GBM,	241	10-fold CV	Not specified	0.870	Accuracy = 94.9%
**Treatment Toxicity Studies**										
Al-Fahad et al. ^25^	ALL	Treatment toxicity prediction	MRI-derived information	Classification of cognitive abilities	LASSO	200	Training (80%) and testing (20%) cohorts used	Not specified	0.870	
Ramalingam et al. ^26^	ALL	Treatment toxicity prediction	Genotypes of SLC19A1, MTHFR, TYMS, and cytogenetic data	Methotrexate-related toxicities	MDR	115	10-fold CV	Not specified		OR = 5.71 -2 Log Likelihood of Reduced Model = 97.104
Zhan et al. ^27^	B-cell ALL	Treatment toxicity prediction	SNPs in 16 genes, clinical characteristics and methotrexate delayed clearance	Predictive models for the risk of neutropenia and fever	RF with ADASYN SVM, DT	139	Training (70%) and testing (30%) cohorts used	Not specified	0.927	
Tram et al. ^28^	Lymphoma	Treatment toxicity prediction	CT image	Risk of treatment-related late effects	NN	100	5-fold CV	Against human raters		Dice value = 0.988 HR = 3.1
Theruvath et al. ^29^	Lymphoma	Dosing prediction	PET/MRI scan data	Enhanced PET/MRI images	NN	20	Not specified	Against neural network called SubtlePET	1	K statistic = 1
**Disease Susceptibility/ Diagnosis Studies**										
Mahmood et al. ^30^	ALL	Classifying risk factors	Clinical, genomic and socio-environmental data	Risk score for ALL	CART, RF, GBM & DT	50	10-fold CV	Not specified		Accuracy = 99.83%
Kulis et al. ^31^	B-Cell Precursor ALL	Classifying risk factors	Antigens measured through flow cytometry	Identification of specific genetic aberrations	GBM	818	5-fold CV	Not specified		OR = 16.90

*ADASYN* Adaptive Synthetic, *ALL* Acute Lymphoblastic Leukaemia, *AML* Acute Myeloid Leukaemia, *AUC* Area Under the Curve, *CART* Classification and Regression Tree, *CV* Cross-validation, *DT* Decision Tree, *GBM* Gradient Boosting Model, *GLM* Generalised Linear Model, *GO* gemtuzumab ozogamicin, *HR* Hazard Ratio, *LncRNA* Long noncoding RNA, *K-NN* k-nearest neighbour, *LASSO* Least Absolute Shrinkage and Selection Operator, *LDA* Linear Discriminant Analysis, *LR* Linear Regression, *MDR* Multifactor Dimensionality Reduction, *NN* Neural Network, *OR* Odds Ratio, *RF* Random Forest, *RHR* Rate of High Risk, *ROR* Rate of Overestimated Risk, *RSR* Rate of Subestimation Risk, *SNP* Single Nucleotide Polymorphism, *SVM* Support Vector Machine

The included studies primarily focused on leukaemia, with specific emphasis on acute lymphoblastic leukaemia (ALL) in 13 studies, acute myeloid leukaemia (AML) in four studies, and an unspecified subtype in 1 study. Additionally, two studies addressed lymphoma. The most commonly used ML methods and algorithms were Random Forest (RF, *n* = 8), Least Absolute Shrinkage and Selection Operator (LASSO, *n* = 6), Gradient Boosting Model (GBM, *n* = 4), and Support Vector Machines (SVM, *n* = 4). Note that multiple papers utilised more than one ML method in a single study.

Cross-validation techniques were employed in 16 (80%) of the studies, including methods such as 5- to 1000-fold cross-validation, leave-one-out cross-validation, training-versus-testing sets, and C-index calculations. External validation was performed in 5 (25%) of all included studies.

### Prognosis and relapse/recurrence studies

Eight studies utilised ML to predict disease outcomes in paediatric haematological malignancies.^12–19^ All studies focused on leukaemia, with five addressing ALL, two on AML, and one on an unspecified subtype. All studies were assessed as “low risk” of bias using the Quality in Prognosis Studies (QUIPS) checklist, which evaluates study participation, attrition, prognostic factor measurement, outcome measurement, study confounding, and statistical analysis,^10^ indicating good overall study quality in this category.

Survival analyses employing genetic data from databases such as TARGET were the most common methodology in this group. For example, one study identified key long non-coding RNAs (LncRNAs) associated with AML prognosis using LASSO Cox analysis, reporting Area Under the Curve (AUC) values of 0.701, 0.704, and 0.696 for 1-, 3-, and 5-year survival, respectively.^15^ AUC values below 0.50 indicate poor predictability, values between 0.51 and 0.70 indicate relatively poor accuracy, values between 0.71 and 0.90 indicate moderate accuracy, and values above 0.90 indicate high accuracy and strong discrimination capability.^32^ Notably, these findings were externally validated using comparative data from The Cancer Genome Atlas (TCGA), although with a lower concordance to the developed model. Overall, four (50%) of the studies in this category were externally validated. For instance, one study using the RF algorithm with 10-fold Monte Carlo cross-validation to predict relapse in ALL achieved an AUC of 0.901. The results were externally validated against an independent test set of 84 patients, demonstrating the robustness and potential clinical applicability of the predictive model. The use of external validation suggests a strong reinforcement of the predictive models’ robustness and applicability in clinical settings. This process, such as using data from TCGA, underscores the potential of these models to generalise across different datasets, enhancing their reliability for clinical prognosis and treatment decision-making in paediatric leukaemia cases.

The studies showed a range of AUC scores from 0.685 to 0.929, indicating a wide variation in model performance. This heterogeneity could be attributed to differences in study design, including varying numbers of patients (range 156–1693) and primary endpoints (e.g., 3-year overall survival vs. 5-year overall survival). Seven (88%) of the studies used AUC as a primary measure of predictive performance. All studies used either LASSO (*n* = 4) or RF (*n* = 3) methods. When grouped by ML method, LASSO models had AUC scores ranging from 0.685 to 0.898, indicating low to moderate accuracy, while RF models had AUC scores ranging from 0.803 to 0.929, indicating moderate to high accuracy. These results suggest that RF techniques may offer marginally superior predictive performance compared to LASSO.

Despite the promise shown by these models, limitations include the use of genetic data from publicly available databases and a lack of relevant paediatric cohort validation. One group of authors highlighted the need for future research to employ more prospective paediatric cohorts due to the limitations associated with using public databases.^15^

In summary, these studies highlight the significant potential of ML methods, particularly RF and LASSO, in predicting disease outcomes in paediatric leukaemia. The variation in AUC scores underscores the importance of strategic ML method selection, reflecting its role in study outcome heterogeneity. These findings highlight the need for a nuanced approach in selecting ML techniques, considering not only AUC scores but also factors like model interpretability and computational demands, to enhance predictive precision in leukaemia prognosis.

### Treatment response studies

Five studies investigated the use of ML to predict treatment response in paediatric haematological malignancies, including three studies on ALL and two on AML.^20–24^ Four of these studies focused on classification tasks. All studies scored 6 or more on the Newcastle-Ottawa Scale (NOS), indicating a generally high standard of methodological quality and reliability in their findings.

In one study, a ten-gene DNA-damage response gene expression signature (CalDDR-GEx10 score) was used to predict responses to gemtuzumab ozogamicin (GO) in paediatric AML patients. The input variables included gene expression levels of 18 genes in DNA-damage response pathways. Patients with high CalDDR-GEx10 scores had lower complete remission (CR) rates and worse event-free survival when treated with GO. This score specifically predicted responses to calicheamicin-induced DNA damage, rather than general chemotherapy effects, with a sensitivity of 72.7%, specificity of 63.6%, and a Positive Predictive Value (PPV) of 61.1%.^20^ Another study employed ML techniques, including k-nearest neighbours (K-NN), SVM, and RF, to RNA sequencing data to predict CR in paediatric AML patients post-induction therapy.^22^ The best result, achieved using a K-NN model with 50 genes, yielded an AUC of 0.812. Both studies were able to predict CR based on genetic data through the utilisation of ML and were cross-validated, highlighting the potential of ML and gene expression signatures in personalised medicine for cancer treatment.

Three (60%) of the studies in this category used AUC as a measure of their models’ ability to predict treatment response, with scores ranging from 0.840 to 0.875, indicating moderate accuracy. Despite the use of different ML algorithms (GBM, K-NN, and Decision Tree), the studies showed similar patient sizes (range 241–473) and endpoints, contributing to low heterogeneity in the evaluation of treatment response prediction. This consistency suggests a reliable evaluation of treatment response prediction across these studies.

However, none of these studies achieved a high accuracy AUC model ( > 0.900), indicating that while the models were moderately effective, they did not reach the threshold of high accuracy. Additionally, none of the studies conducted external validation, which limits the clinical utility of these models. Prospective studies with external validation are needed to assess the impact of these ML models on treatment decision-making and patient outcomes. Despite these limitations, the findings support the potential of ML to enhance personalised medicine in this field.

### Treatment toxicity studies

ML was used to predict adverse treatment effects in five studies.^25–29^ Three studies focused on ALL and two on lymphoma. The Newcastle-Ottawa Scale (NOS) was used to assess the quality of these studies, with all scoring six or more, indicating high methodological quality.

One study explored the relationship between genetic variations and treatment-related adverse effects (TRAEs) in paediatric patients with ALL undergoing methotrexate therapy. It found a significant association between the SLC19A1 (c.80 G > A) genotype and increased TRAEs, with an odds ratio (OR) of 5.71 (*p* < 0.01). Multinomial logistic regression and multifactor dimensionality reduction analysis supported this association, confirming the genotype’s strong correlation with TRAEs.^26^ Another study also focused on methotrexate therapy, using ML to predict neutropenia and fever associated with high-dose methotrexate treatment in paediatric B-ALL. The best model, using a combined RF with Adaptive Synthetic (ADASYN) resampling, achieved an AUC of 0.870 to 0.927, sensitivity of 0.916–0.935, and specificity of 0.920–0.924.^27^

In another study, CT images were used to predict late TRAEs. A deep learning model demonstrated high concordance with manual human analysis, evidenced by Dice scores greater than 0.950 and a K-statistic of 1.00. Notably, once trained, the model segmented body composition from CT datasets in under a second, highlighting the potential of ML models to rapidly and accurately process extensive datasets. Validated against external manual analysis, this model shows promise for clinical application due to its capability to deliver rapid and reliable results.^28^

The studies varied widely in their statistical analyses, making it difficult to comment on heterogeneity. Only two studies used AUC as a measure of effect. These AUC values were 0.870 (moderate accuracy) and 0.927 (high accuracy), suggesting strong predictive capabilities of ML models in this context.

The primary limitation of these studies is the lack of uniform reporting of effect measures, which hampers the ability to review heterogeneity and draw robust conclusions. Additionally, the sample sizes in these studies (range 20 to 200) were smaller compared to other categories, limiting the statistical power to detect significant associations. Moreover, translating these findings into clinical practice requires validation in larger, multi-centre studies to confirm their utility in predicting treatment-related toxicities. Only one of these studies included external validation, underscoring the need for further validation efforts.

### Others: disease susceptibility & diagnosis studies

This review identified two studies focused on developing predictive models for disease susceptibility and diagnostics in paediatric haematological malignancies.^30,31^ These studies provide insights into the early application of ML in identifying risk factors and diagnostic markers.

One study employed several ML algorithms, including Classification and Regression Tree (CART), RF, GBM, and C5.0 decision tree, to identify key attributes influencing ALL susceptibility.^30^ Platelet count was identified as a crucial predictor, and the CART algorithm demonstrated a high model accuracy of 99.8%. However, this study lacked external validation, which limits the generalisability of the findings and highlights the need for further investigation in more varied and larger cohorts.

Similarly, the second study within this group also utilised ML models for disease susceptibility but did not perform external validation. The lack of validation is a significant limitation as it prevents the confirmation of the models’ applicability in different clinical settings. Despite this, the preliminary findings suggest that ML can identify important predictive factors for disease susceptibility.

Overall, while the limited number of studies prevents a comprehensive thematic analysis, these findings indicate the potential of ML in enhancing early disease detection and risk assessment in paediatric haematological cancers. The absence of external validation across both studies underscores the need for further research to ensure the reliability and practical utility of these ML models.

## Discussion

The review reveals a promising trend of ML models achieving moderate to high accuracy across the examined thematic categories. ML methods such as RF and LASSO have emerged as effective tools in paediatric haematological malignancies, as reflected in their prevalence across the studies reviewed. These studies demonstrate a strong emphasis on predictive tasks, highlighting a growing interest in using ML for prognosis and treatment outcome prediction. Most research thus far lies in prognosis models, with further research warranted in diagnosis and treatment toxicity prediction models. An adequate number of studies exist in treatment response studies. It is crucial, however, to assess the real-world applicability of these findings through external validation, considering the diverse methodologies and sample sizes across studies.^33,34^

The lack of external validation in many studies is a significant limitation that prevents the replication and generalisation of ML models across different datasets. The heterogeneity of the data collected, including variations in patient populations, data sources, and ML methodologies, complicates the replication of these models. To address this issue, future studies should focus on standardising data collection methods and reporting metrics. The need for standardised reporting guidelines, such as the Transparent Reporting of a multivariable prediction model for Individual Prognosis or Diagnosis (TRIPOD),^35^ is highlighted in a similar review of the literature.^36^ Moreover, the limited clinical deployment of ML algorithms, with many studies showing limited clinical applicability, is a common criticism.^37–39^

This review also illustrates the infancy of ML application in this field, marked by the limited number of studies included in most thematic categories identified. The initial literature search yielded over 20 abstract reports that, despite being excluded due to not meeting inclusion criteria, indicate a growing application of ML in paediatric haematology with vast use across the field. For example, a study using Prediction Analysis of Microarrays (PAM) to identify paediatric patients with B-ALL with a Ph-like signature for better clinical intervention employed ML on gene expression profiles from 811 patients, leading to a 15-gene classifier that showed high sensitivity (93.0%) and specificity (89.7%) in tests.^40^ The classifier was also able to identify genomic lesions linked to Ph-like ALL, associated with poor clinical outcomes. The findings suggest that integrating this classifier in clinical practice could help identify patients for targeted therapy, potentially improving treatment outcomes. The numerous abstracts noted in the literature search highlight the rapidly growing application of ML in paediatric haematological cancers. As these datasets grow, they offer new opportunities for applying novel ML approaches, potentially transforming the field.

The review highlights the potential of ML to enhance patient care by providing clinicians and health professionals with data-driven insights that can inform diagnostic and treatment decisions. While promising, the integration of ML in clinical practice should support, not replace, healthcare providers. For instance, ML algorithms have achieved 97.0% accuracy in identifying leukaemia from peripheral blood smears, thereby supporting clinical investigations.^41^ Furthermore, the integration of ML-enhanced technologies such as the Countess 3 Automated Cell Counter in bone marrow transplant labs exemplifies a shift toward more precise and efficient diagnostic processes, showing notable improvements over traditional manual methods.^42,43^ This trend is supported by a previous reporting of methodology that calls for strong evaluation frameworks to measure the actual impact of ML on patient outcomes, thus ensuring its role as a complement to, not a replacement for, clinical decision-making.^44^

Comparing findings between paediatric and adult/mixed studies reveals key insights. In adult/mixed cohorts, ML applications show significant improvements with AUC ranges of 0.71 – 0.93 for prognosis/relapse prediction,^45,46^ 0.85 – 0.97 for treatment response,^22,47,48^ and 0.59 – 0.90 in toxicity predictions.^49,50^ These studies have a superior AUC for prognosis/ relapse and treatment response predictions as compared to the paediatric cohort. The superior AUC in adult models highlights their robustness, likely due to larger sample sizes and more extensive datasets. Adult studies may also benefit from standardised methodologies and larger, diverse cohorts, contributing to increased generalisability. Conversely, paediatric studies face challenges such as smaller sample sizes and heterogeneous designs, leading to broader AUC ranges and reduced generalisability.

The scope of this review is narrowed by the predominance of studies focused on leukaemia, specifically ALL and AML, with only two studies extending to non-leukemic haematological malignancies. This lack of diversity within the spectrum of paediatric haematological cancers limits our capacity to generalise the findings of ML across the broader field. Consequently, while our review suggests substantial advancements in the ML-driven management of leukaemia, the translatability of these insights to other haematological conditions remains to be ascertained. With only a small number of studies employing external validations, we are unable to comment on the feasibility of implementing these ML algorithms in the current clinical setting. This underscores an imperative for future research to encompass a wider range of haematological disorders, thus enhancing the robustness and clinical relevance of ML prognostic, diagnostic, and treatment response models in paediatric haematology.

Addressing these challenges of methodological heterogeneity and limited clinical deployment is crucial for the implementation of ML in paediatric malignancies.^51^ The expanding datasets in this domain offer an opportunity for applying novel ML approaches. However, increased standardisation in study designs and reporting standards, like the TRIPOD guidelines mentioned above, is essential to achieve this. Future research should focus on prospective studies and fostering interdisciplinary collaboration to develop and implement clinically relevant ML tools. Moreover, integrating ML with clinical workflows and validating these models in diverse, real-world settings will be vital in ensuring their practical utility and improving outcomes for children with cancer.

## Conclusion

This systematic review highlights the growing role of ML in paediatric haematological malignancies, demonstrating its potential to significantly enhance diagnostic accuracy, prognostic predictions, and treatment strategies. Despite moderate to high accuracy achieved by ML models, the clinical applicability remains constrained due to the lack of external validation and methodological heterogeneity. Addressing these challenges through larger, diverse datasets, standardised reporting, and robust external validation is crucial for translating ML from a promising research tool into a reliable component of clinical practice. This advancement could lead to more precise and personalised treatment approaches, ultimately improving outcomes for children with cancer.

## Supplementary information

Search Strategy
