# Supplementary material for: Machine learning in paediatric haematological malignancies: a systematic review of prognosis, toxicity and treatment response models
Source: Pediatr Res. 2024 Aug 31;97(2):524–31. doi: 10.1038/s41390-024-03494-9 (PMC12014474; doi:10.1038/s41390-024-03494-9)
Supplement: Supplementary file 1 — Search Strategy [file 41390_2024_3494_MOESM1_ESM.pdf]

#	SEARCH TERM	NO. OF RESULTS
1	exp Leukemia/ or leukemia*.mp. or leukaemia.mp	965471
2	exp Lymphoma/ or lymphoma*.mp.	799022
3	exp Myeloma/ or myeloma*.mp.	218389
4	exp Artificial intelligence/ or exp Machine Learning/ or “deep learning”.mp. or “neural network”.mp. or “predictive model”.mp.	955059
5	exp Pediatrics/ or pediatric*.mp. or paediatric*.mp. or “child*”.mp.	7347333
6	((chemotherapy adj3 (toxic or side effect*)) or (treatment adj3 (failure or success or response or outcome)) or (prognosis or survival or relapse or remission)).mp.	8918841
7	exp Hematologic Neoplasms/ or hematologic* neoplasm*.mp. or haematologic*neoplasm*.mp.	3143821
8	1 or 2 or 3	1721284
9	7 or 8	4029772
10	4 and 5 and 6 and 9	711
